# Predicting Long-Term Recovery of Consciousness in Prolonged Disorders of Consciousness Based on Coma Recovery Scale-Revised Subscores: Validation of a Machine Learning-Based Prognostic Index

**DOI:** 10.3390/brainsci13010051

**Published:** 2022-12-27

**Authors:** Alfonso Magliacano, Piergiuseppe Liuzzi, Rita Formisano, Antonello Grippo, Efthymios Angelakis, Aurore Thibaut, Olivia Gosseries, Gianfranco Lamberti, Enrique Noé, Sergio Bagnato, Brian L. Edlow, Nicolas Lejeune, Vigneswaran Veeramuthu, Luigi Trojano, Nathan Zasler, Caroline Schnakers, Michelangelo Bartolo, Andrea Mannini, Anna Estraneo

**Affiliations:** 1 IRCCS Fondazione Don Carlo Gnocchi ONLUS, 50143 Firenze, Italy; 2 Polo Specialistico Riabilitativo, Fondazione Don Carlo Gnocchi, 83054 Sant’Angelo dei Lombardi, Italy; 3 Scuola Superiore Sant’Anna, Istituto di BioRobotica, 56025 Pontedera, Italy; 4 Fondazione Santa Lucia IRCCS, 00179 Rome, Italy; 5 Neurosurgery Department, University of Athens Medical School, 11527 Athens, Greece; 6 Coma Science Group, GIGA Consciousness-University and University Hospital of Liège-Liège-Belgium, 4000 Liège, Belgium; 7 Neurorehabilitation and Vegetative State Unit E. Viglietta, 12100 Cuneo, Italy; 8 IRENEA-Instituto de Rehabilitación Neurológica, Fundación Hospitales Vithas, 46011 Valencia, Spain; 9 Unit of Neurophysiology and Unit for Severe Acquired Brain Injuries, Rehabilitation Department, Giuseppe Giglio Foundation, 90015 Cefalù, Italy; 10 Center for Neurotechnology and Neurorecovery, Department of Neurology, Massachusetts General Hospital, Boston, MA 02114, USA; 11 CHN William Lennox, 1340 Ottignies, Belgium; 12 Division of Clinical Neuropsychology, Thomson Hospital Kota Damansara, Petaling Jaya 47810, Malaysia; 13 Department of Psychology, University of Campania L. Vanvitelli, 81100 Caserta, Italy; 14 Concussion Care Centre of Virginia, Ltd., Richmond, VA 23233, USA; 15 Research Institute, Casa Colina Hospital and Centers for Healthcare, Pomona, CA 91767, USA; 16 Neurorehabilitation Unit, HABILITA Zingonia/Ciserano, 24040 Bergamo, Italy

**Keywords:** disorders of consciousness, coma recovery scale-revised, prognosis, rehabilitation, machine learning

## Abstract

Prognosis of prolonged Disorders of Consciousness (pDoC) is influenced by patients’ clinical diagnosis and Coma Recovery Scale-Revised (CRS-R) total score. We compared the prognostic accuracy of a novel Consciousness Domain Index (CDI) with that of clinical diagnosis and CRS-R total score, for recovery of full consciousness at 6-, 12-, and 24-months post-injury. The CDI was obtained by a combination of the six CRS-R subscales via an unsupervised machine learning technique. We retrospectively analyzed data on 143 patients with pDoC (75 in Minimally Conscious State; 102 males; median age = 53 years; IQR = 35; time post-injury = 1–3 months) due to different etiologies enrolled in an International Brain Injury Association Disorders of Consciousness Special Interest Group (IBIA DoC-SIG) multicenter longitudinal study. Univariate and multivariate analyses were utilized to assess the association between outcomes and the CDI, compared to clinical diagnosis and CRS-R. The CDI, the clinical diagnosis, and the CRS-R total score were significantly associated with a good outcome at 6, 12 and 24 months. The CDI showed the highest univariate prediction accuracy and sensitivity, and regression models including the CDI provided the highest values of explained variance. A combined scoring system of the CRS-R subscales by unsupervised machine learning may improve clinical ability to predict recovery of consciousness in patients with pDoC.

## 1. Introduction

After a coma due to severe acquired brain injury, patients can persist in prolonged (>28 days) Disorders of Consciousness (pDoC) [1,2]. The prolonged states generally include the vegetative state/unresponsive wakefulness syndrome (VS/UWS) [3], characterized by wakefulness without awareness and reflexive albeit not purposeful behavioral responses, as well as the minimally conscious state (MCS) [4], in which patients can manifest minimal and inconsistent but reproducible voluntary behaviors.

The pDoC can persist chronically, but some patients have the potential to progress to full consciousness. The management of such complex patients requires an expert multi-disciplinary team [5,6]. In this context, clinicians are routinely called upon to provide the patient’s family with prognostic information for decision-making and interventional planning purposes, the latter including intensity and duration of treatment [7]. Bilateral absence of the N20 cortical component on somatosensory evoked potentials is the most robust predictor of poor outcome, particularly in patients in coma and pDoC due to hypoxic-ischemic/anoxic injury [8]. Unfortunately, N20 responses are rarely recorded in the post-acute phase. A clinical diagnosis of MCS due to traumatic brain insult has been found to be a predictor of a more favorable clinical outcome relative to VS/UWS likely in relation to a less severe brain damage and to a higher level of cognitive awareness/responsiveness [9,10,11]. 

Moreover, bedside neurobehavioral measures such as the Coma Recovery Scale-Revised (CRS-R) [12] total score have been found to provide high prognostic accuracy for long-term outcomes [8,13,14,15,16]. The CRS-R is the most reliable and validated clinical tool for distinguishing patients in MCS from those in VS/UWS [12,17]. It consists of 23 items organized in six subscales assessing patients’ behavioral responses independently from each other on auditory, visual, motor, oromotor, communication, and arousal functions. A higher score in a single subscale can classify the patient as being in MCS or as emerging from MCS (i.e., eMCS, patients who have recovered full consciousness and demonstrate functional communication and/or functional object use) [4]. By considering only the highest subscore for the diagnostic classification of patients in MCS or eMCS, the other domains evaluated by the CRS-R that describe patients’ characteristics are usually neglected. Nonetheless, it has been shown that a composite score obtained by considering the highest CRS-R subscore on every subscale can improve diagnostic accuracy [18]. 

Recently, a Consciousness Domain Index (CDI), computed by means of unsupervised machine learning through the combination of the CRS-R subscores, classified a large cohort of patients with pDoC into two clusters, where the visual and motor subscales were the most discriminating factors. The clustering predicted patients who showed consciousness recovery at 6 months post-injury with a higher accuracy than the clinical diagnosis [19]. The prognostic value of the CDI has only been tested in a single patient sample for predicting a short-term outcome (i.e., 6 months post-injury). The current study goals included: a) externally validating the prognostic accuracy of the CDI, with respect to patient clinical diagnosis and to CRS-R total score; and b) investigating the prediction accuracy of the CDI on long-term consciousness recovery at 6-, 12-, and 24-months post-injury.

## 2. Materials and Methods

### 2.1. Study Design and Patient Samples

This retrospective analysis was conducted on data from a multicenter perspective study launched by the International Brain Injury Association Disorders of Consciousness-Special Interest Group (IBIA DoC-SIG), aimed at examining the clinical evolution of a large sample of patients with pDoC as well as identifying outcome prognostic factors for these patients [14,15].

The IBIA DoC-SIG database [14] was used for the external validation of the CDI, previously internally validated on a different database from an Italian prospective study (hence “reference database”) [6,19]. For both studies, inclusion criteria were: (i) age ≥ 18 years; (ii) clinical diagnosis of VS/UWS or MCS, according to standard diagnostic criteria [4]; (iii) traumatic or non-traumatic (i.e., vascular or hypoxic-ischemic/anoxic) etiology; and (iv) time post-injury from 28 days to 3 months. Exclusion criteria were: (i) previous history of acquired brain injury, psychiatric, or neurodegenerative disease; and (ii) coexisting neoplasms, severe organ dysfunction, or unstable clinical condition (e.g., hemodynamic instability or severe respiratory failure) that could directly impact the outcome. In both studies, each center collected patient demographic data (i.e., age, sex) and information about medical history (i.e., etiology and time post-injury at study entry). Within 1 week from study entry, repeated CRS-R assessments (at least three times within a 1-week period) were performed for all patients to confirm the clinical diagnosis. The CRS-R with the best total score was considered for the statistical analysis. 

In the reference database, patients were followed-up at 6 months post-injury; whereas in the IBIA DoC-SIG study, patients were followed up at 6-, 12-, and 24-months post-injury. In both databases, the clinicians at the participating centers assessed patient consciousness level and clinical diagnosis by means of CRS-R during hospital stay or, after discharge, at home or in chronic care facilities. In the present study, the primary outcome was the recovery of full consciousness (i.e., patients in VS/UWS or MCS who progressed to eMCS) at 6-, 12-, and 24-months post-injury.

The demographic and clinical characteristics of patients included in the IBIA DoC-SIG database were compared with those of patients in the reference database. Numerical independent variables such as age, time post-injury, CRS-R total score, and sub-scores were compared by means of t-test or Mann–Whitney U test, as appropriate after normality tests. For categorical independent variables as sex, etiology, and clinical diagnosis, χ^2^ tests were performed.

### 2.2. Cluster Estimation and External Validation

Patients from the reference database were used to derive cluster properties and to deploy the unsupervised model as internally validated in a previous methodological study [19]. In that study, the number of clusters (N_clusters_) that best divided the cohort was 2 (yielding maximal Silhouette score). In the present study, we applied the following procedure:

(i). Gathering the CRS-R subscores of each patient in the reference database.
(ii). Estimating centroids with partitional clustering algorithms (K-means++ clustering, 500 random initializations) [20] for each training fold of a five-fold cross-validation split.
(iii). Applying a *twin-sample* validation approach to each validation set [21] which involved conducting both the cluster training and the validation phases on the training as well as the validation sets and obtaining two cluster labels for each sample. The two different sets of labels for the validation data were compared achieving the twin-validation accuracy for each validation fold. These metrics allowed us to check the stability of the clustering process.
(iv). Aggregating twin-validation accuracies across folds in order to obtain a k-fold cross-validated twin-sample accuracy. The centroids from the fold resulting in the minimum twin-validation error were employed.
(v). Assigning each patient in the external validation set (IBIA DoC-SIG database) to the cluster with minimum 6-dimensional Euclidean distance between her/his CRS-R subscores and the two cluster centroids. Thus, the assignment to a specific cluster (CDI = 0 or CDI = 1) represents the CDI of that patient.

The CDI derived for the patients in the external validation set (IBIA DoC-SIG database) was compared with the clinical diagnosis and the CRS-R total score. In order to appropriately compare the features of the CDI and of the CRS-R total score, the latter was dichotomized based on the two cut-offs that have been found to provide the highest diagnostic accuracy (93%; CRS-R total score = 8), and the highest specificity for identifying MCS or eMCS (100%; CRS-R total score = 10) [22]. The CDI derived for the patients in the external validation set was compared with:

(i). The clinical diagnosis at study entry (i.e., VS/UWS vs. MCS).
(ii). A binary CRS-R total score using 8 as cut-off (hence CRS-R_8_).
(iii). A binary CRS-R total score using 10 as cut-off (hence CRS-R_10_)

The comparisons were performed by means of contingency tables and χ^2^ analyses with recovery of full consciousness at 6-, 12-, and 24-months post-injury as outcome. For each analysis, χ^2^ values and univariate accuracy were estimated, as well as sensitivity and specificity. 

### 2.3. Multivariate Analysis

We investigated the relationships between five independent variables (i.e., CDI, clinical diagnosis, CRS-R_8_, CRS-R_10_, and the CRS-R total score) and the outcome at 6-, 12-, and 24-month post-injury by means of multivariate logistic regressions, for a total of 15 regression models (five set of independent variables × 3 follow-ups). Confounding variables included in the regression models from study entry as patients’ age, sex, time post-injury, and etiology. The Nagelkerke pseudo-R^2^, which indicates the amount of variability accounted for by the predictors in each regression model, was compared across models. For evaluating the discrimination ability of the logistic regression models, the area under the curve of the receiver operating characteristic curve (AuROC) was also computed.

## 3. Results

### 3.1. Cohort Comparison

The IBIA DoC-SIG database included 143 patients for whom clinical diagnosis was available at each follow-up (i.e., 6, 12, and 24 months after brain injury). The study flow-chart and details on baseline characteristics of included patients are reported elsewhere [14,15]. 

Patients in the IBIA DoC-SIG database were found to be significantly younger than patients in the reference database (*p* = 0.003) and showed a significantly longer time post-injury (*p* < 0.001; Table 1). Etiologies were represented in significantly different proportions between the two databases (χ^2^ = 10.701(1); *p* = 0.013). In particular, Bonferroni-corrected pairwise z-tests revealed a significantly higher percentage of patients with other etiologies (different than traumatic, vascular, or hypoxic-ischemic/anoxic) in the reference database (corrected-*p* < 0.05). No significant differences were found between the two databases regarding sex, clinical diagnosis at study entry, CRS-R total score and subscores, and percentages of patients who recovered full consciousness at six-month follow-up (*p* > 0.05; Table 1). 

### 3.2. Cluster Estimation and Validation

In order to select clusters for external validation, the centroids producing minimum twin-validation error were retained for further analysis, thus resulting in the ones deriving from the third fold (see Table 2). Either CDI, the clinical diagnosis, or the binary CRS-R scores were found to be associated with the outcome at 6, 12, and 24 months (all *p* < 0.001), with a higher χ^2^ value for all follow-ups when the CDI was adopted (Table 3). 

**Figure 1:**
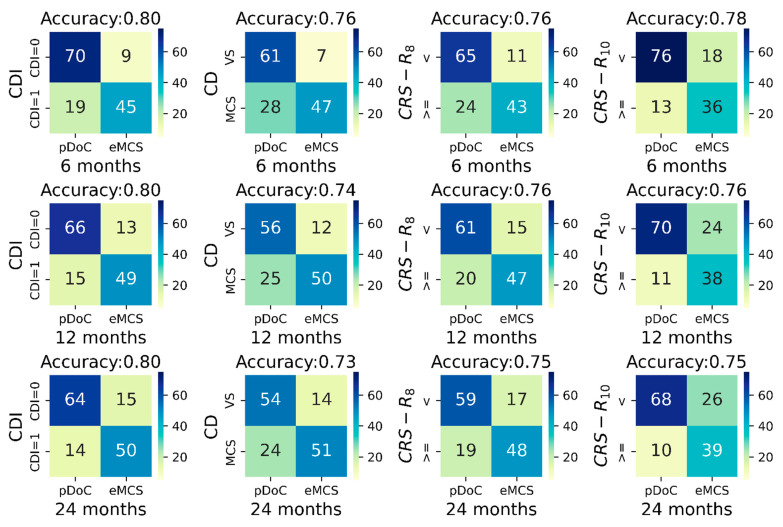
Contingency tables between CDI, clinical diagnosis at study entry, CRS-R_8_, CRS-R_10_ (columns left to right) with respectively the outcome at 6 months (top row), at 12 months (middle row), and at 24 months (bottom row). Each contingency table has on the x-axis the outcome set as presence/absence of a pDoC at the different outcome timing, and on the y-axis patients with CDI = 1 or CDI = 0 (first column), patients in VS or in MCS (second column), and patients with CRS-R smaller than (<) or greater/equal to (>=) the indicated threshold (third and fourth columns). Colors indicate the relative amount of patients within each cell; the color gradient is reported within the color bar on the right of each table. Abbreviations: CD, clinical diagnosis; CDI, Consciousness Domain Index; CRS-R, Coma Recovery Scale-Revised; eMCS, emergence from the minimally conscious state; pDoC, prolonged disorder of consciousness; VS, vegetative state.

The univariate prediction accuracy of the CDI was stable across the follow-ups (80%), whereas a slight decrease in accuracy was found as a function of the follow-up time using the clinical diagnosis, or the CRS-R_8_ and the CRS-R_10_ (see Figure 1). 

Importantly, the CDI univariate prediction accuracy was higher by 4%, 6%, and 7% for the 6-, 12-, and 24-month outcome, respectively, compared to accuracy of the clinical diagnosis. Similarly, the prediction accuracy of the CDI was also higher than that of the CRS-R_8_ and of the CRS-R_10_ (see Figure 1).

The CDI classification improved the sensitivity of predicting recovery of full consciousness at 6 months post-injury from 68.5% to 78.6% (~10%) with a decrease in specificity of ~3%, compared to clinical diagnosis. Similarly, at 12- and 24-months post-injury, the sensitivity of the CDI vs. clinical diagnosis was 81.5% (specificity = 79%) vs. 69.1% (specificity = 80.6%), and 82% (specificity = 76.9%) vs. 69.2% (specificity = 78.5%), respectively. 

As expected, the CRS-R_10_ provided a higher sensitivity for identifying patients who recovered full consciousness with respect to CRS-R_8_ (Figure 1). 

### 3.3. Multivariate Analysis

The CDI, the clinical diagnosis, the CRS-R_8_, the CRS-R_10_, and the CRS-R total score were all associated significantly with the outcome at 6, 12, and 24 months after the brain injury (all *p* < 0.001). In all multivariate logistic regressions, a shorter time post-injury was found to be an indicator of higher likelihood of recovery of full consciousness (*p* < 0.05; Table 4). 

In the regression model including the CDI and in the model using the CRS-R total score, a younger age was significantly associated with a good outcome at all follow-ups (*p* < 0.05), whereas hypoxic-ischemic/anoxic (vs. non-hypoxic-ischemic/anoxic) etiology to brain injury was associated with a poorer outcome at 12- and 24-month post-injury (*p* < 0.05). In the regression models including the clinical diagnosis, a younger age and male sex were significantly associated with a good outcome at 12-, 24-, and 6-months (*p* < 0.05) ), respectively. A younger age and male sex were also significantly associated with a good outcome at all follow-ups (*p* < 0.05) in the regression model including the CRS-R_8_. In the models including the CRS-R_10,_ a younger age was associated with good outcome at 12 and 24 months (*p* < 0.05).

The explained variance of the predictors (R^2^) and the AuROC were found to be higher when using the CDI than either the clinical diagnosis or the CRS-R total score (see Table 4).

## 4. Discussion

Accurately identifying patients with pDoC with higher likelihood of recovery of consciousness is crucial when providing prognostic information to patient’s relatives, other practitioners and payors, as well as for planning tailored care and rehabilitation pathways. Prognostication of patients with pDoC remains a major challenge due to a lack of validated outcome predictors. This multicenter international study confirmed that classifying individual patients by means of a novel clinical index (i.e., the CDI) combining the CRS-R subs-cores improved prognostic accuracy compared to clinical diagnosis or to the CRS-R total score, as previously observed in a large cohort of patients with pDoC [19]. 

Notwithstanding the differences in age, time post-injury and etiology between the present and the original validation databases, the prediction accuracy obtained by means of the CDI was stable at 80%. Importantly, the improvement of the prediction accuracy obtained by means of the CDI, compared to the clinical diagnosis, was found to be stable at approximately 5%, with a sensitivity 10% higher than for clinical diagnosis. This finding suggests that the prediction value of the CDI observed previously [19] was in fact not impacted by patient demographics and medical history but was strictly related to the behavioral findings. The CDI derives from a simultaneous multi-dimensional assessment of consciousness, balancing items derived from all CRS-R subscales. The CDI could therefore be considered an index of the level of behavioral response complexity in the multiple domains assessed by CRS-R subscales, thus allowing to stratify patients with pDoC. 

Further cross-sectional studies are needed to investigate the possible correlation between CDI and functional connectivity in networks associated with processing of cognitive functions evaluated by the CRS-R subscales. Moreover, in line with a previous study [19], in the present cohort of patients the visual, auditory, and motor CRS-R subscales were the most crucial factors to differentiate the two centroids of the CDI clusters. The visual subscale in particular was found to affect dissimilarity among clusters. This finding could likely be ascribed to the main role of the CRS-R visual subscale in detecting early signs of consciousness [5,23].

Confirming the prediction accuracy of the CDI on a multicenter external cohort of patients (i.e., IBIA DoC-SIG database) allows us to validate the prediction model’s reproducibility and generalizability and to propose its use in different clinical and geographic locales as well as cohorts of patients with pDoC [24,25]. 

Additionally, in the univariate analysis, the prediction accuracy of the CDI was higher than that of the two values (i.e., 8 and 10) of the CRS-R total score, which have been found to be crucial for recognizing patients in VS/UWS (CRS-R total score < 8) and patients with minimal or full consciousness (CRS-R total score ≥ 10) [22]. This finding further supports the hypothesis that the higher predictive value of the CDI could be explained by the fact that the CDI considers information from single subscores, assigning a weight to each sub-scale, whereas the CRS-R total score can denote the overall level of responsiveness to multisensory stimulations [12].

The CDI, clinical diagnosis, and CRS-R total score, together with younger age, shorter time post-injury, male sex, and non-hypoxic-ischemic/anoxic brain injury, were independent predictors of recovery of full consciousness at a multivariate level. A younger age has been frequently associated with a higher likelihood of a good outcome in patients with pDoC [8,14,16,26], probably due to better general health conditions [27] and to a greater level of potential neuroplasticity [28]. A higher degree of brain plasticity is also usually associated with focal brain injuries (i.e., vascular or traumatic), in which spared cortical areas can support functional reorganization [29]. The association between clinical outcome and male sex might be ascribed to sex-related differences in patient care [30], as care of male patients tends to be more frequently supported by caregivers than care of female patients [15,31,32]. In addition, a shorter time after injury at study entry (i.e., at admission to an early care program for patients with pDoC) might characterize patients who spent less time in intensive care units, likely due to less severe brain injury and less medical and neurosurgical complications in the acute phase [14,33,34].

Finally, we found that the improved prediction accuracy at a multivariate level of the CDI with respect to clinical diagnosis and CRS-R continuous and binary CRS-R total scores was also confirmed at short (6 months), long (12 months), and very long (24 months) assessment points after the initial brain insult, suggesting that the composite of higher behaviors significantly impacted the probability of recovery of consciousness, regardless of the time after injury.

There were several study limitations worth noting. First, the analysis could not be carried out, targeting the minimal improvement from UWS/VS to MCS, which has been considered as an outcome in previous longitudinal studies [14] showing the high prognostic relevance of the CRS-R total score. However, the present study aimed to externally validate a behavioral index of consciousness (i.e., CDI) with the already described high predictive value of full recovery of consciousness [19]. Second, the CDI in the IBIA DoC-SIG database is quite consistent with the clinical diagnosis as the majority of patients in VS/UWS (64 out of 68) were assigned a CDI of 0 and the majority of patients in MCS (60 out of 75) were assigned a CDI of 1. Similarly, in the reference database [19], agreement between CDI and clinical diagnosis was found to be 94% (Cohen’s k = 0.85). This substantial overlap may suggest that CDI may be redundant, as the index closely parallels the clinical diagnosis. However, we found a 5% increase in prognostic accuracy and 10% increase in sensitivity compared with clinical diagnosis, which may further improve clinical ability to identify patients with a high probability of recovery of full consciousness. Furthermore, we could not investigate the prognostic value of CDI within each diagnostic group (i.e., VS/UWS and MCS), evaluating patients for which the CDI and the clinical diagnosis differed, due to the small sample size. However, the CDI is a novel index that could be applied for stratifying patients with pDoC regardless of clinical diagnosis. Further cohort studies are needed for evaluating the ability of CDI to profile for prognosis even patients with covert consciousness in whom making an accurate clinical diagnosis based on behavioral responses is challenging because of severe cognitive and motor impairments [35]. 

Finally, we could not evaluate the medical complications that have been found to negatively impact clinical outcomes of patients with DoC [36,37]. However, we believe this innovative and combined measure of behavioral responses could lead to improved prognostic profiling of this challenging patient population with pDoC regardless of the specific clinical diagnosis. 

## 5. Conclusions

Patients in MCS (vs. in VS/UWS) and with a higher CRS-R total score usually show a better clinical outcome at both short- and long-term, but the prediction accuracy of the clinical diagnosis and of the CRS-R total score remains limited. The present validation study demonstrated that the CDI, an unsupervised machine-learning clinical index based on a combination of the different functions assessed by the CRS-R subscales, can improve prediction accuracy and sensitivity for consciousness recovery at 6, 12, and 24 months after brain injury.

## Figures and Tables

**Table 1:** Demographic, anamnestic, and clinical characteristics at baseline of included patients in the reference and validation databases.

	Reference (*N* = 190)	IBIA DoC-SIG (*N* = 143)	*p*
Age, years	58.5 (21.6)	53 (35)	**0.001**
Sex, M	130 (68.4)	102 (71.3)	0.568
Time post-injury, days	39 (33)	56 (54)	**<0.001**
Etiology			**0.013**
TBI	65 (34.2)	55 (38.5)	--
HI/A	33 (17.4)	35 (24.5)	--
Vascular	82 (43.2)	53 (37.1)	--
Other	10 (5.2)	0 (0)	--
Diagnosis, MCS	97 (51.1)	75 (52.4)	0.801
CRS-R, total score	7 (6)	7 (7)	0.927
Auditory	1 (1)	1 (2)	0.645
Visual	1 (3)	1 (3)	0.607
Motor	2 (2)	2 (4)	0.306
Oro-motor	1 (0)	1 (1)	0.057
Communication	0 (0)	0 (0)	0.099
Arousal	2 (1)	2 (1)	0.991
6-month outcome, eMCS	86 (45.3)	54 (37.8)	0.170
12-month outcome, eMCS	--	62 (43.4)	--
24-month outcome, eMCS	--	65 (45.5)	--

Descriptive data are reported as median (inter-quartile range) for continuous and ordinal variables, and as counts of patients for each level of categorical variables (percentage), i.e., sex, etiology, diagnosis, and outcomes. Univariate statistics are based upon the t-test, Mann–Whitney U test, or χ^2^ tests, as appropriate. Significant *p*-values are reported in bold. Abbreviations: CRS-R, Coma Recovery Scale-Revised; eMCS, emergence from the MCS; HI/A, hypoxic-ischemic/anoxic; M, male; MCS, minimally conscious state; TBI, traumatic brain injury.

**Table 2:** Cluster centroids for each cross-validation fold and related medians and IQR.

Fold n	Au	V	M	OM	C	AR
CDI = 1						
1	2.42	2.56	3.26	1.51	0.47	1.95
2	2.50	2.74	3.22	1.56	0.44	2.02
**3**	**2.33**	**2.65**	**3.37**	**1.37**	**0.46**	**1.98**
4	2.41	2.72	3.45	1.36	0.50	2.00
5	2.27	2.60	3.33	1.47	0.51	1.91
Median	2.41	2.65	3.33	1.47	0.47	1.98
IQR	0.09	0.12	0.11	0.14	0.04	0.05
CDI = 0						
1	0.85	0.58	1.28	0.66	0.01	1.31
2	0.82	0.55	1.41	0.69	0.03	1.32
**3**	**0.81**	**0.54**	**1.24**	**0.66**	**0.01**	**1.28**
4	0.91	0.61	1.22	0.70	0.01	1.34
5	0.89	0.54	1.21	0.73	0.01	1.30
Median	0.85	0.55	1.24	0.69	0.01	1.31
IQR	0.07	0.04	0.06	0.04	0.00	0.02

In bold, the fold which resulted in minimum twin-validation error, thus the centroids retained for further analysis. Abbreviations: AU, auditory; AR, arousal; C, communication; CDI, Consciousness Domain Index; IQR, inter-quartile range; M, motor; OM, oromotor; V, visual.

**Table 3:** Univariate chi-square analysis between CDI, clinical diagnosis, CRS-R_8_, and CRS-R_10_ with the outcome (recovery of full consciousness) at different follow-ups for the IBIA DoC-SIG database. Degrees of freedom are equal to one for all table entries (i.e., 2 x 2 contingency tables).

	CDI	Clinical Diagnosis	CRS-R_8_	CRS-R_10_
6 months	χ^2^ = 52.226(1), *p* < 0.001	χ^2^ = 41.623(1), *p* < 0.001	χ^2^ = 37.432(1), *p* < 0.001	χ^2^ = 39.268(1), *p* < 0.001
12 months	χ^2^ = 52.013(1), *p* < 0.001	χ^2^ = 34.895(1), *p* < 0.001	χ^2^ = 36.859(1), *p* < 0.001	χ^2^ = 34.461(1), *p* < 0.001
24 months	χ^2^ = 49.873(1), *p* < 0.001	χ^2^ = 32.335(1), *p* < 0.001	χ^2^ = 34.869(1), *p* < 0.001	χ^2^ = 34.050(1), *p* < 0.001

Abbreviations: CDI, Consciousness Domain Index; CRS-R, Coma Recovery Scale-Revised.

**Table 4:** Multivariate logistic regressions for predicting recovery of full consciousness at 6, 12, and 24 months after brain injury. Panels A, B, C, D, and E indicate regressions including the CDI, the clinical diagnosis, the CRS-R_8_, the CRS-R_10_, and the CRS-R total score, respectively. First, second, and third columns refer to the analysis targeting 6-, 12- and 24- months outcomes, respectively.

A	R^2^ = 0.621, AuROC = 0.902			R^2^ = 0.619, AuROC = 0.898			R^2^ = 0.645, AuROC = 0.907		
	OR	95% CI	*p*	OR	95% CI	*p*	OR	95% CI	*p*
Age	**0.967**	** 0.935–0.999 **	** 0.044 **	** 0.951 **	** 0.920–0.983 **	** 0.003 **	** 0.936 **	** 0.904–0.970 **	** <0.001 **
Sex (F)	0.359	0.116–1.112	0.076	0.345	0.113–1.050	0.061	0.305	0.097–0.954	0.051
TPI	** 0.971 **	** 0.956–0.987 **	** <0.001 **	** 0.976 **	** 962–991 **	** 0.002 **	** 0.977 **	** 0.962–0.992 **	** 0.003 **
Etiology (TBI)	0.927	0.246–3.833	0.968	0.338	0.084–1.363	0.127	0.344	0.082–1.442	0.145
Etiology (HI/A)	0.510	0.131–1.984	0.331	0.208	0.053–0.817	0.025	0.179	0.043–0.735	0.017
CDI = 1	** 16.699 **	** 11.288–119.310 **	** <0.01 **	** 32.740 **	** 10.694–100.234 **	** <0.001 **	** 35.892 **	** 11.027–116.821 **	** <0.001 **
**B**	**R^2^ = 0.595, AuROC = 0.897**			**R^2^ = 0.519, AuROC = 0.866**			**R^2^ = 0.539, AuROC = 0.876**		
	**OR**	**95% CI**	***p***	**OR**	**95% CI**	***p***	**OR**	**95% CI**	***p***
Age	0.975	0.945–1.007	0.126	** 0.964 **	** 0.937–0.993 **	** 0.014 **	** 0.953 **	** 0.925–0.982 **	** 0.002 **
Sex (F)	** 0.322 **	** 0.106–0.978 **	** 0.046 **	0.368	0.135–1.004	0.051	0.347	0.127–0.949	0.039
TPI	** 0.963 **	** 0.946–0.980 **	** <0.001 **	** 0.973 **	** 0.959–0.987 **	** <0.001 **	** 0.974 **	** 0.960–0.988 **	** <0.001 **
Etiology (TBI)	1.963	0.514–7.496	0.320	0.721	0.216–2.411	0.595	0.743	0.220–2.511	0.633
Etiology (HI/A)	0.490	0.131–1.839	0.291	** 0.256 **	** 0.075–0.880 **	** 0.031 **	** 0.241 **	** 0.069–0.837 **	** 0.025 **
Diagnosis (MCS)	** 32.109 **	** 10.753–142.243 **	** <0.001 **	** 16.431 **	** 5.866–46.025 **	** <0.001 **	** 15.079 **	** 5.366–42.376 **	** <0.001 **
**C**	**R^2^ = 0.573, AuROC =0.890**			**R^2^ = 0.558, AuROC = 0.882**			**R^2^ = 0.588, AuROC = 0.891**		
	**OR**	**95% CI**	***p***	**OR**	**95% CI**	***p***	**OR**	**95% CI**	***p***
Age	** 0.967 **	** 0.937–0.999 **	** 0.041 **	** 0.954 **	** 0.924–0.984 **	** <0.001 **	** 0.940 **	** 0.910–0.972 **	** <0.001 **
Sex (F)	** 0.336 **	** 0.114–0.990 **	** 0.049 **	** 0.326 **	** 0.114–0.931 **	** 0.036 **	** 0.294 **	** 0.101–0.857 **	** 0.025 **
TPI	** 0.964 **	** 0.946–0.980 **	** <0.001 **	** 0.969 **	** 0.955–0.984 **	** <0.001 **	** 0.970 **	** 0.955–0.985 **	** <0.001 **
Etiology (TBI)	1.487	0.408–5.417	0.547	0.554	0.155–1.978	0.363	0.554	0.150–2.045	0.375
Etiology (HI/A)	0.525	0.141–1.953	0.336	** 0.234 **	** 0.064–0.858 **	** 0.028 **	** 0.206 **	** 0.054–0.781 **	** 0.020 **
CRS-R_8_ (≥8)	** 29.554 **	** 8.900–98.145 **	** <0.001 **	** 23.186 **	** 7.629–70.471 **	** <0.001 **	** 24.577 **	** 7.730–78.147 **	** <0.001 **
**D**	**R^2^ = 0.550, AuROC = 0.880**			**R^2^ = 0.510, AuROC = 0.865**			**R^2^ = 0.556, AuROC = 0.886**		
	**OR**	**95% CI**	***p***	**OR**	**95% CI**	***p***	**OR**	**95% CI**	***p***
Age	0.973	0.945–1.002	0.070	** 0.960 **	** 0.932–0.988 **	** 0.006 **	** 0.946 **	** 0.917–0.976 **	** <0.001 **
Sex (F)	0.512	0.180–1.456	0.209	0.486	0.180–1.313	0.155	0.425	0.151–1.191	0.104
TPI	** 0.970 **	** 0.955–0.984 **	** <0.001 **	** 0.976 **	** 0.963–0.989 **	** <0.001 **	** 0.976 **	** 0.963–0.990 **	** <0.001 **
Etiology (TBI)	1.809	0.515–6.352	0.355	0.669	0.200–2.245	0.515	0.661	0.190–2.307	0.517
Etiology (HI/A)	0.633	0.166–2.414	0.503	0.278	0.077–1.007	0.051	** 0.238 **	** 0.061–0.923 **	** 0.038 **
CRS-R_10_ (≥10)	** 22.585 **	** 7.607–67.052 **	** <0.001 **	** 15.808 **	** 5.767–43.438 **	** <0.001 **	** 19.324 **	** 6.518-57.296 **	** <0.001 **
**E**	**R^2^ = 0.608, AuROC = 0.895**			**R^2^ = 0.589, AuROC = 0.890**			**R^2^ = 0.612, AuROC = 0.901**		
	**OR**	**95% CI**	***p***	**OR**	**95% CI**	***p***	**OR**	**95% CI**	***p***
Age	** 0.967 **	** 0.937–0.998 **	** 0.035 **	** 0.953 **	** 0.923–0.984 **	** 0.003 **	** 0.940 **	** 0.909–0.972 **	** <0.001 **
Sex (F)	0.503	0.171–1.476	0.211	0.461	0.163–1.301	0.144	0.410	0.142–1.182	0.099
TPI	** 0.961 **	** 0.944–0.978 **	** <0.001 **	** 0.967 **	** 0.952–0.983 **	** <0.001 **	** 0.968 **	** 0.952–0.984 **	** <0.001 **
Etiology (TBI)	1.757	0.480–6.427	0.395	0.601	0.170–2.120	0.428	0.596	0.164–2.158	0.430
Etiology (HI/A)	0.635	0.160–2.524	0.519	** 0.254 **	** 0.064–0.998 **	** 0.050 **	** 0.220 **	** 0.053–0.909 **	** 0.036 **
CRS-R total	** 1.503 **	** 1.298–1.741 **	** <0.001 **	** 1.467 **	** 1.277–1.685 **	** <0.001 **	** 1.477 **	** 1.278–1.708 **	** <0.001 **

Significant *p*-values are reported in bold. Abbreviations: AuROC, area under the receiver operating curve; CI, confidence intervals; CDI, Consciousness Domain Index; CRS-R, Coma Recovery Scale-Revised; F, female; HI/A, hypoxic-ischemic/anoxic; MCS, minimally conscious state; OR, odds ratios; TBI, traumatic brain injury; TPI, time post-injury.

## Data Availability

The data presented in this study are available on request from the corresponding author.
